# A Hybrid VMD-Transformer-BiLSTM Framework with Cross-Attention Fusion for Aileron Fault Diagnosis in UAVs

**DOI:** 10.3390/s26072256

**Published:** 2026-04-06

**Authors:** Yang Song, Weihang Zheng, Xiaoyu Zhang, Rong Guo

**Affiliations:** School of Intelligent Science and Technology, Beijing University of Civil Engineering and Architecture, Beijing 100044, China; 201906040222@stu.bucea.edu.cn (W.Z.); zhangxiaoyu@bucea.edu.cn (X.Z.); guorong@bucea.edu.cn (R.G.)

**Keywords:** unmanned aerial vehicle (UAV), fault diagnosis, variational mode decomposition (VMD), Transformer, bidirectional long short-term memory (BiLSTM)

## Abstract

**Highlights:**

**What are the main findings?**

- A hybrid VMD-Transformer-BiLSTM framework is proposed for aileron fault diagnosis in fixed-wing UAVs by identifying distinctive fault features in residual signals. The proposed method achieves 95.12% accuracy for fixed-wing UAV aileron fault diagnosis, outperforming existing methods.
- A cross-attention mechanism is designed by combining the Transformer and BiLSTM networks to integrate the global and local fault features for improving the diagnosis performance.

**What are the implications of the main findings?**

- The proposed VMD-Transformer-BiLSTM framework provides a reliable scheme for the aileron fault diagnosis in fixed-wing UAVs, with potential applications in flight safety monitoring and predictive maintenance.
- The cross-attention mechanism utilizes selective focus on discriminative features, indicating potential for superior performance compared to single-network architectures, highlighting the significance of complementary feature fusion.

**Abstract:**

Aileron fault diagnosis in fixed-wing unmanned aerial vehicles (UAVs) faces significant challenges due to strong noise, multi-modal coupling, and limited fault samples. This paper presents a hybrid fault diagnosis framework that integrates variational mode decomposition (VMD) with a cross-attention-based feature fusion mechanism. First, residual signals are generated from UAV kinematic models and decomposed into multi-scale intrinsic mode functions (IMFs) using VMD to extract multiscale frequency-localized features. An integrated framework is then constructed, where Transformer encoders capture the global features and bidirectional long short-term memory (BiLSTM) networks extract local temporal dynamics. To effectively combine these complementary features, a cross-attention fusion module is designed to focus on the discriminative time-frequency features. Furthermore, a hybrid pooling strategy integrating max pooling and attention pooling is introduced to enhance classification robustness. Experiments on the AirLab failure and anomaly (ALFA) dataset demonstrate that the proposed method achieves 95.12% accuracy with improved fault separability, outperforming VMD + BiLSTM (87.66%), VMD + Transformer (86.89%), Transformer + BiLSTM (84.83%), Transformer (72.24%), CNN + LSTM (94.05%), and HDMTL (94.86%).

## 1. Introduction

Due to the autonomous flight capabilities, unmanned aerial vehicles (UAVs) have become valuable tools in various industries, including agriculture, environmental monitoring, logistics, and military reconnaissance [1,2]. However, the complexity of the UAV flight control system, the constantly changing flight environment, and component aging can lead to faults, such as actuator jamming and structural damage [3,4]. These faults not only decrease the accuracy of the UAV’s flight control, but can also result in accidents and crashes. Therefore, it is essential to conduct fault diagnosis for UAV flight control systems to enhance the safety and reliability of UAVs and ensure mission execution.

Existing research on UAV fault diagnosis can be broadly divided into model-based and data-driven methods. Model-based methods utilize UAV dynamics and kinematic models to design observers or filters for residual generators, and rely on predefined thresholds for diagnosis results [5,6]. Specifically, a simplified third-order Thau observer was employed for adaptive estimation of actuator faults while maintaining system stability [6]. Similarly, an observer-based residual was designed using the $H_{-}/L_{\infty }$ optimization problem for the linear parameter-varying models of quadrotor UAVs; the residual evaluation is achieved by analyzing directional correlations between the residual signal and fault feature vector [7]. These approaches are effective when accurate analytical models of UAVs are available and noise levels are low. However, fixed-wing UAVs exhibit strong nonlinearities and operate in noisy environments, and these factors significantly degrade the diagnosis performance. To effectively tackle the challenges of nonlinearities and uncertainties in model-based methods, both adaptive observers [8] and sliding mode techniques [9] were used for improved diagnosis performance. However, these approaches still heavily depend on precise system models and predetermined thresholds, which restrict their ability to adapt to changing flight conditions and levels of noise.

Data-driven methods have attracted significant attention due to their ability to extract features directly from historical and real-time data, without relying on explicit analytical models [10,11]. For instance, a deep reinforcement learning strategy was applied for the adaptive identification of actuator faults in multirotor UAVs [10]. Additionally, Li et al. [11] combined a learnable wavelet packet transform (WPT) with graph-based label enhancement for multirotor fault diagnosis. However, most studies have primarily focused on multirotor UAVs and sensor faults, leaving the diagnosis of actuator faults in fixed-wing UAVs relatively unexplored.

Due to aging components, as well as interference from airflow and noise, unmanned aerial vehicles are inevitably prone to malfunctions. These faults can be broadly categorized into actuator faults, sensor faults, and structural faults. Actuator faults include aileron jamming, elevator malfunction, rudder fault, and propeller or motor damage, which directly affect the control surfaces and thrust generation. Sensor faults involve faults in inertial measurement units (IMUs), GPS, and pitot tubes, leading to incorrect state estimation. Structural faults refer to damage to the airframe, wings, or control surfaces, often caused by fatigue, collisions, or environmental stress. Among actuator faults, aileron faults are critical for fixed-wing UAVs, as they directly impair roll control and can lead to loss of stability. This paper focuses on aileron jamming faults, including left aileron jamming, right aileron jamming, and dual aileron jamming, as well as the fault-free case.

Diagnosing aileron faults in fixed-wing UAVs presents unique challenges that distinguish it from other fault diagnosis tasks. One difficulty is the mechanical symmetry between the left and right ailerons, resulting in similar vibration transmission paths and moment responses that can cause confusion in identifying fault features. Additionally, strong operational noise and model uncertainty introduce multi-modal coupling in residual signals, making it difficult to isolate fault signals among multiple frequency components. Furthermore, a severe scarcity of fault samples due to safety constraints makes it challenging to train deep learning models. To address aileron fault detection, Qin et al. [12] utilized fuzzy entropy to determine optimal sliding window sizes and proposed a multi-branch network structure to adapt to feature loss at low sampling rates. In addition, He et al. [13] proposed a generative adversarial learning framework to reconstruct missing signals, with a reconstruction module designed to enhance the generalization performance of the detection model. However, these studies primarily focused on fault detection rather than fault isolation, which is a more challenging task.

To summarize, many existing UAV fault diagnosis methods are designed for multirotor UAVs or sensor faults, and only limited attention is given to actuator faults in fixed-wing UAVs. Furthermore, the presence of strong operational noise and model uncertainty, combined with the symmetry between the left and right ailerons, makes fault diagnosis particularly challenging. Many existing hybrid models also lack effective fusion strategies to integrate different features, which limits the diagnosis performance. These challenges have prompted the need for a feature-fused diagnosis framework specifically for aileron faults in fixed-wing UAVs.

The UAV signals exhibit strong non-stationarity and multi-modal coupling, making signal decomposition a critical preprocessing step for effective fault diagnosis. Among the commonly used methods, empirical mode decomposition (EMD) suffers from mode mixing and lacks a solid theoretical foundation, making it unreliable for distinguishing mechanically symmetric left and right aileron faults. Additionally, wavelet transform (WT) offers multi-resolution analysis but its performance heavily depends on the empirical selection of wavelet basis functions with limited adaptability to varying fault characteristics. In contrast, VMD formulates signal decomposition as a constrained variational problem, providing mathematical rigor and analytical formulation [14]. Unlike the iterative sifting process of EMD, VMD extracts all modes simultaneously through joint optimization, enabling better error balancing between modes. With concentrated center frequencies, VMD allows proper single-mode decomposition and facilitates the distinction between left and right aileron faults with similar vibration transmission paths. VMD has proven to be a powerful tool for deep learning models to more effectively learn fault-specific features [15,16], and has shown promise in fault diagnosis applications [17,18]. Therefore, VMD is selected to effectively separate the multi-modal coupled aileron fault residuals into IMFs, providing discriminative inputs for the subsequent feature extraction framework.

In addition to signal decomposition, deep learning architectures have also evolved significantly in fault diagnosis to capture specific features from sequential data. Long short-term memory (LSTM) and bidirectional LSTM (BiLSTM) networks are known for effectively capturing temporal dependencies through gated structures [19]. On the other hand, the Transformer architecture, with its self-attention mechanism, has shown strong capability in capturing global dependencies across entire sequences and has been explored for fault diagnosis in UAV sensors [20].

However, no single architecture can effectively capture both global dependencies and local temporal dynamics. For aileron fault diagnosis, both global and local features are equally important, making a hybrid approach necessary. To achieve this, feature fusion strategies have been proposed to combine complementary representations from multiple architectures, which have been successfully applied in mechanism fault diagnosis [21]. Zhang et al. [22] extracted the temporal and spatial features for UAVs using CNN and LSTM networks, and proposed the heterogeneous deep multi-task learning (HDMTL) scheme using an attention-based adaptive sharing strategy to improve the diagnosis accuracy. Particularly, the combination of VMD, Transformer, and BiLSTM has shown superior performance in remaining life prediction [23], demonstrating the framework’s ability to extract abnormal features. This work motivated the application of this framework in aileron fault diagnosis to extract discriminative features between left and right aileron faulty cases while suppressing irrelevant information from disturbances and noise.

Despite the widespread adoption of VMD, Transformer and BiLSTM architectures in fault diagnosis, the direct application to aileron fault identification in fixed-wing UAVs remains limited. The combination of these methods remains challenging due to the mechanical symmetry between left and right ailerons, strong noise, and multi-modal coupling. To address these challenges, this paper proposes a fault diagnosis framework that integrates VMD, Transformer, BiLSTM, and cross-attention fusion for aileron faults in fixed-wing UAVs. The VMD on residuals provides robustness to the diagnosis model in tackling multi-modal coupling. To capture complementary global and local features, Transformer and BiLSTM are combined, and a cross-attention mechanism is designed to enable selective focus on discriminative features for distinguishing between left and right aileron faults, despite their mechanical symmetry. By integrating these components, the proposed framework aims to achieve accurate aileron fault diagnosis under challenging conditions of strong noise, limited samples, and symmetric fault patterns. The key contributions of this study can be summarized as follows:

(1) The proposed framework utilizes both Transformer and BiLSTM to extract global dependencies and local temporal dynamics from the IMFs generated by performing the VMD on the residual signal. These features associated with aileron faults provide a more comprehensive analysis of the fault diagnosis performance.
(2) A cross-attention module is introduced for feature fusion, selectively amplifying discriminative patterns for symmetric mechanical faults. The fused features are then improved through a combination of max pooling and attention pooling, resulting in enhanced diagnosis performance.
(3) The proposed method integrates residual, VMD-based IMFs, and deep feature learning into a unified framework, enabling automatic fault diagnosis without the need for manual thresholding.

## 2. The Proposed Framework

**Figure 1:**
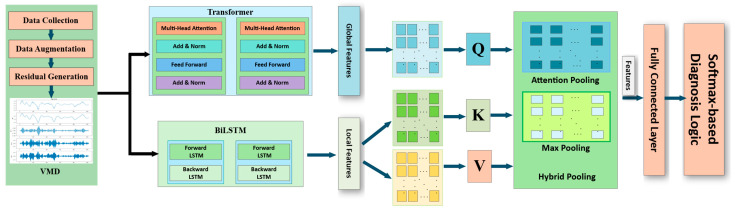
The overall fault diagnosis model for aileron faults in fixed-wing UAVs.

This paper focuses on the aileron fault diagnosis for fixed-wing UAVs. In the absence of sufficient faulty data, a data augmentation scheme is used. The residual is generated, which is then subjected to VMD. For feature learning, both the Transformer encoders and BiLSTM are introduced to extract features, followed by a cross-attention mechanism and a hybrid pooling strategy for feature fusion. The overall framework is shown in Figure 1.

### 2.1. Data Preprocessing

Existing data-driven fault diagnosis models rely on a large amount of balanced, high-quality data under normal operation and fault scenarios. However, it is often difficult to obtain sufficient fault samples for UAV flight control systems. If insufficient data is used directly for training, the diagnosis scheme will become sensitive to noise or changes under normal operations, resulting in poor fault diagnosis performance.

To address these issues, a common approach is to introduce data augmentation methods. Compared to strong augmentation methods, mild augmentation significantly improves the learning stability of models in small-sample and noisy environments while preserving the physical characteristics of the original signals. In this paper, we employ a mild time-series data augmentation strategy, which involves applying amplitude and noise perturbation to the original signals, simulating fluctuations during operation.

### 2.2. Residual Generation

Consider the flight attitude model of the UAVs as follows:

(1) $$\left\{\begin{array}{l} \dot{\phi }=\omega_{x}+\tan\theta (\omega_{y}\sin\phi +\omega_{z}\cos\phi )\\ \dot{\theta }=\omega_{y}\cos\phi -\omega_{z}\sin\phi \\ \dot{\psi }=(\omega_{y}\sin\phi +\omega_{z}\cos\phi )/\cos\theta \end{array}\right.$$

where $\omega_{x}$, $\omega_{y}$, and $\omega_{z}$ are the angular velocities in body axes, ϕ, θ, and ψ stand for the roll, pitch, and yaw angles in relation to the vehicle-carried Earth axes and body axes, respectively.

Define the state as $x(t)={\left[\begin{array}{cccccc} \omega_{x}(t) & \omega_{y}(t) & \omega_{z}(t) & \phi (t) & \theta (t) & \psi (t) \end{array}\right]}^{T}$. The control input consists of throttle settings and control surface deflections, denoted by u(t), including the left and right aileron deflection, denoted by $\delta_{l}$ and $\delta_{r}$, respectively. Based on the mechanism analysis of UAVs, preliminary aileron faults affect the roll angular channel in the UAV flight control systems. Using the discretization and linearization methods, we obtain

(2) $$\left\{\begin{array}{l} \phi (k+1)=a_{\phi }x(k)+b_{\phi }u(k)+e_{\phi ,f_{1}}f_{1}(k)+e_{\phi ,f_{2}}f_{2}(k)+w(k)\\ \phi_{m}(k)=\phi (k)+v(k) \end{array}\right.$$

where ϕ(k) denotes the actual roll angle at the time step k, $\phi_{m}(k)$ is the measured value for ϕ(k), which can be obtained using a gyroscope and accelerometer. w(k),v(k) represent the disturbance and noise, $f_{1}$ represents the left aileron faulty case, $f_{2}$ is the right aileron faulty case. The fault-free case is denoted as $f_{0}$ with $f_{1}=0,f_{2}=0$ and the dual aileron faulty scenario is denoted as $f_{3}$ with $f_{1}\neq 0,f_{2}\neq 0$. $a_{\phi },b_{\phi },e_{\phi ,f_{1}},e_{\phi ,f_{2}}$ are known vectors generated from the UAV model.

The expected signal for the roll angle, denoted as $\hat{\phi }(k)$, can be estimated using the following Kalman filter:

(3) $$\hat{\phi }(k)=a_{\phi }\hat{x}(k-1)+b_{\phi }u(k-1)+l(k-1)(\phi_{m}(k-1)-\hat{\phi }(k-1))$$

where l(k−1) is the gain vector for the Kalman filter.

Define the residual as follows:

(4) $$r(k)=\phi_{m}(k)-\hat{\phi }(k)$$

It is obvious from Equations (2) and (4) that the residual contains the fault, disturbance, and noise information. However, the mechanical symmetry of UAVs, strong noise, and model uncertainty introduce multi-modal coupling in the signal r(k), making it difficult to identify the left, right, and dual aileron faulty scenarios. Additionally, due to the safety constraints of UAVs, there is a scarcity of aileron fault samples, resulting in undesirable performance of diagnosis models. These challenges motivate a new aileron fault diagnosis framework for fixed-wing UAVs. Throughout the rest of this paper, the residual signal r(k) in the roll angular channel will be utilized for diagnosing the fault-free case $f_{0}$, left aileron faulty case $f_{1}$, right aileron faulty case $f_{2}$, and dual aileron faulty scenario $f_{3}$. in fixed-wing UAV flight control systems.

### 2.3. Variational Mode Decomposition

Due to the multi-modal coupling of the left and right aileron in residuals, the direct application of residuals in the fault diagnosis model can hardly guarantee the diagnosis performance. Therefore, the residual signal is further processed using variational mode decomposition (VMD) to separate it into multiple IMFs, each capturing different frequency components. All the IMFs serve as the input to the deep learning model, which automatically learns fault-specific features and maps them to the corresponding fault categories. The diagnosis is not based on a simple threshold, but on a data-driven feature learning and classification process that captures subtle differences between fault types. The VMD on the residual can be performed as follows:

(5) $$r(k)=\sum_{h=1}^{H}v_{h}(k)=\sum_{h=1}^{H}A_{h}(k)\cos(\varphi_{h}(k))$$

where H is the total number of IMFs, $v_{h}(k)$ for h=1,2,⋯,H represents the H IMFs after VMD, $A_{h}(k)$ denotes the instantaneous amplitude of $v_{h}(k)$, and $\varphi_{h}(k)$ denotes the instantaneous phase of $v_{h}(k)$.

The parameter H can be set based on preliminary center-frequency analysis. Aileron faults exhibit complex signatures that span across multiple frequency bands. Manually discarding any IMF might lead to a loss of subtle fault characteristics, so we use all the IMFs for the cross-attention mechanism to dynamically assign adaptive weights to different features.

### 2.4. Global-Local Feature Fusion Mechanism

While the Transformer excels at extracting global dependencies through the self-attention mechanism, it often lacks sensitivity to local information. The BiLSTM is effective at modeling short-term dependencies, but its performance degrades with longer sequences. Therefore, diagnosis models relying on a single network struggle to simultaneously capture global and local features. To address these limitations, a hybrid diagnosis model is proposed by integrating both the Transformer and BiLSTM. This model utilizes a cross-attention fusion mechanism to effectively combine the respective strengths of each network in feature extraction, resulting in improved diagnosis performance.

#### 2.4.1. Global Feature Extraction Based on Transformer

With the global attention mechanism, the Transformer provides an effective tool for extracting global features. It is composed of encoder and decoder components. Each encoder is designed with an identical structure but different weight coefficients, including a self-attention layer and a feed-forward network layer. Here, we focus on using the Transformer encoders to extract the global dependency features from IMFs $v_{h}(k)$. Perform linear embedding on $v_{h}(k)$ yields

(6) $$X_{T}=\left[\begin{array}{cccc} v_{1}(T) & v_{1}(T+N_{h}) & \ldots & v_{1}(T+(L-1)N_{h})\\ v_{2}(T) & v_{2}(T+N_{h}) & \ldots & v_{2}(T+(L-1)N_{h})\\ \;\;\;\vdots & \vdots & \ddots & \vdots \\ v_{H}(T) & v_{H}(T+N_{h}) & \ldots & v_{H}(T+(L-1)N_{h}) \end{array}\right]$$

where $X_{T}\in {\mathbb{R}}^{H\times L}$, $L,\;N_{h}$ are the linear embedding parameters.

In the implementation, we set the stride $N_{h}=1$ to retain the full temporal resolution, and the sequence length L=1024, and all the IMFs are used as input.

For the Transformer, the multi-head self-attention mechanism computes attention scores for each head. For the n-th attention head (n=1,2,…,N), the query $Q_{T}^{\left(n\right)}\in {\mathbb{R}}^{L\times d_{k}}$, key $K_{T}^{\left(n\right)}\in {\mathbb{R}}^{L\times d_{k}}$, value $V_{T}^{\left(n\right)}\in {\mathbb{R}}^{L\times d_{v}}$ matrices are obtained by linear projections:

(7) $$Q_{T}^{\left(n\right)}=X_{T}W_{TQ}^{\left(n\right)},\;K_{T}^{\left(n\right)}=X_{T}W_{TK}^{\left(n\right)},\;V_{T}^{\left(n\right)}=X_{T}W_{TV}^{\left(n\right)}$$

where $W_{TQ}^{\left(n\right)}\in {\mathbb{R}}^{H\times d_{k}},W_{TK}^{\left(n\right)}\in {\mathbb{R}}^{H\times d_{k}}$, and $W_{TV}^{\left(n\right)}\in {\mathbb{R}}^{H\times d_{v}}$ are the learnable query, key, and value projection matrices, $d_{k}$ is the dimension of the query and key vectors, $d_{v}$ is the dimension of the value vectors, and we set $d_{k}=d_{v}=L/N$.

The attention output for n-th attention head is computed as follows:

(8) $$H_{n}=\mathrm{softmax}\left(\frac{Q_{T}^{\left(n\right)}{\left(K_{T}^{\left(n\right)}\right)}^{⊤}}{\sqrt{d_{k}}}\right)V_{T}^{\left(n\right)}$$

where $H_{n}\in {\mathbb{R}}^{H\times d_{v}}$, ${\left(K_{T}^{\left(n\right)}\right)}^{⊤}$ represents the transposed matrix of $K_{T}$, softmax is applied row-wise to normalize attention weights.

The outputs from all N attention heads are concatenated through a linear layer:

(9) $$F_{T}=\mathrm{Concat}\left(H_{1},H_{2},\ldots ,H_{N}\right)W_{O}$$

where $F_{T}\in {\mathbb{R}}^{H\times L}$, $W_{O}\in {\mathbb{R}}^{(N\cdot d_{v})\times L}$ is an output projection matrix.

At each time step, a position-wise feed-forward network is implemented to perform the following nonlinear transformation:

(10) ZT=FFNFT=ReLUFTW1+b1W2+b2

where $Z_{T}\in {\mathbb{R}}^{H\times L}$, $W_{1}\in {\mathbb{R}}^{H\times d_{ff}}$ and $W_{2}\in {\mathbb{R}}^{H\times d_{ff}}$ are the weight matrices, $b_{1}$ and $b_{2}$ are the bias parameters, and ReLU is the activation function, $d_{ff}$ is the hidden layer dimension of the feedforward network.

By stacking multiple encoder layers, the Transformer model effectively captures global dependencies from $X_{T}$, allowing for the extraction of global features $Z_{T}$.

#### 2.4.2. Local Feature Extraction Based on BiLSTM

Parallel to the Transformer, the IMFs are also utilized as the input signal for a BiLSTM to conduct modeling on each modal signal. By using the BiLSTM, the final hidden state representation for each time step is obtained from both the forward and backward directions. Define $x_{T}={\left[\begin{array}{cccc} v_{1}(T) & v_{2}(T) & \cdots & v_{H}(T) \end{array}\right]}^{T}$, and capture the local features using

(11) $$\vec{h_{T}}=LSTM_{f}\left(x_{T},\vec{h_{T-1}}\right)$$

(12) $$\overset{\leftarrow }{h_{T}}=LSTM_{b}\left(x_{T},\overset{\leftarrow }{h_{T-1}}\right)$$

(13) $$h_{T}=\left[\begin{array}{cc} \vec{h_{T}} & \overset{\leftarrow }{h_{T}} \end{array}\right]$$

where $\vec{h_{T}},\overset{\leftarrow }{h_{T}}\in {\mathbb{R}}^{d_{lstm}},d_{lstm}$ is the hidden layer dimension for each direction, $h_{T}\in {\mathbb{R}}^{2d_{lstm}}$ represents the local temporal features at time step T, containing information from both forward and backward temporal dependencies.

The hidden states at each time step form the following temporal feature matrix:

(14) $$H_{B}=\left[h_{1},h_{2},\ldots ,h_{L}\right]\in {\mathbb{R}}^{2d_{\mathrm{lstm}}\times L}$$

The transpose of $H_{B}$ satisfies $H_{B}^{⊤}\in {\mathbb{R}}^{L\times 2d_{\mathrm{lstm}}}$. This matrix $H_{B}^{⊤}$ effectively captures the dynamic features of the IMFs at a local temporal scale. By introducing the local temporal encoding of the BiLSTM, the model provides short-term dependency information.

#### 2.4.3. Cross-Attention-Based Feature Fusion Mechanism

To effectively fuse the output from the Transformer $Z_{T}\in {\mathbb{R}}^{H\times L}$ with the output from BiLSTM $H_{B}^{⊤}\in {\mathbb{R}}^{L\times 2d_{\mathrm{lstm}}}$, we define

(15) $$Q=Z_{T}W_{Q}\in {\mathbb{R}}^{H\times d_{k}},\;K=H_{B}^{⊤}W_{K}\in {\mathbb{R}}^{L\times d_{k}},\;V=H_{B}^{⊤}W_{V}\in {\mathbb{R}}^{L\times d_{v}}$$

where $W_{Q}\in {\mathbb{R}}^{L\times d_{k}},W_{K}\in {\mathbb{R}}^{2d_{lstm}\times d_{k}},W_{V}\in {\mathbb{R}}^{2d_{lstm}\times d_{v}};$ $d_{k},d_{v}$ the same values are selected for the multi-head of the Transformer. The query Q is computed from the Transformer’s global features $Z_{T}$, while the key K and value V are computed from BiLSTM’s local features H. $W_{Q}$, $W_{K}$, and $W_{V}$ are the learnable weights of the cross-attention.

The output of single-head cross-attention can be obtained using

(16) $$F_{\mathrm{cross},i\;}=\mathrm{softmax}\left(\frac{QK^{⊤}}{\sqrt{d_{k}}}\right)V\in {\mathbb{R}}^{H\times d_{v}}$$

Adopting a N head mechanism and linearly projecting all heads after concatenation, the fused features are obtained as follows:

(17) $$F_{fused\;}=\left[\begin{array}{cccc} F_{cross,1\;} & F_{cross,2\;} & \cdots & F_{cross,N\;} \end{array}\right]\in {\mathbb{R}}^{H\times L}$$

By using the BiLSTM temporal features and Transformer features, the fusion model focuses on key time-frequency features, and achieves complementarity of global and local features through the cross-attention mechanism. The result $F_{fused\;}$ can provide more discriminative features for subsequent fault classification.

#### 2.4.4. Hybrid Pooling and Classification Decision

After performing cross-attention fusion, this section utilizes a pooling structure to compress the temporal features into fixed-length vectors for further fault diagnosis. However, relying on a single pooling strategy can result in insufficient information. While max pooling can extract the strongest activation response for each feature and capture key fault features, attention pooling is better suited for global classification by assigning adaptive weight to the most effective features. The output of attention pooling layer is

(18) $$f_{\mathrm{attn}}=\sum_{h=1}^{H}\alpha_{h}F_{\mathrm{fused}}[h,:]$$

where adaptive weight $\alpha =\left[\begin{array}{cccc} \alpha_{1} & \alpha_{2} & \cdots & \alpha_{H} \end{array}\right]=\mathrm{softmax}\left(F_{\mathrm{fused}}q\right)$, $q\in {\mathbb{R}}^{L}$ is the learnable query vector.

The output of the maximum pooling is

(19) $$f_{\max}=\max_{h=1,\ldots ,H}F_{\mathrm{fused}\;}[h,:]\;$$

To provide a more comprehensive analysis of the features, a hybrid parallel pooling structure is proposed. This structure combines the outputs of both max pooling and attention pooling, defined as follows:

(20) $$f_{\mathrm{pool}\;}=\left[f_{\mathrm{attn}\;}\;f_{\max}\right]\in {\mathbb{R}}^{2L}$$

The feature vector $f_{\mathrm{pool}\;}$ is then fed into a fully-connected classifier, and the predicted probability for each faulty case via the following Softmax function:

(21) $$\hat{y}=\mathrm{softmax}\left(f_{\mathrm{pool}}W_{c}+b_{c}\right)\in {\mathbb{R}}^{4}$$

where $W_{c}\in {\mathbb{R}}^{2L\times 4}$ and $b_{c}\in {\mathbb{R}}^{4}$ are trainable parameters, the result $\hat{y}$ contain the probability of corresponding fault-free case, left aileron faulty case, right aileron faulty case, and dual aileron faulty case.

By employing a cross-attention fusion strategy, the fault diagnosis system can effectively preserve the most discriminative features before fault classification, ultimately enhancing the overall fault diagnosis performance. The design of the UAV aileron fault diagnosis system can be summarized as Algorithm 1.
**Algorithm 1.** The VMD-Transformer-BiLSTM-based fault diagnosis model algorithmInput: UAV model, VMD parameter H, max training epochs E. Output: Trained fault diagnosis model. Step 1: Collect UAV flight data and augment the training set via mild noise perturbation. Step 2: Generate residual r(k) using expected and measured roll angles. Step 3: Decompose r(k) into H IMFs $v_{1}(k),v_{2}(k),\cdots ,v_{H}(k)$ using VMD. Step 4: for each training epoch from 1 to E do Step 5: Construct $X_{T}$ using $v_{1}(k),v_{2}(k),\cdots ,v_{H}(k)$, and put $X_{T}$ into the Transformer encoders to extract global temporal features $Z_{T}$. Step 6: Construct $x_{T}$ using $v_{1}(k),v_{2}(k),\cdots ,v_{H}(k)$, and put $x_{T}$ into the BiLSTM layers to extract local temporal features $H_{B}$. Step 7: Set $Q=Z_{T}W_{Q}\in {\mathbb{R}}^{H\times d_{k}},\;K=H_{B}^{⊤}W_{K}\in {\mathbb{R}}^{L\times d_{k}},\;V=H_{B}^{⊤}W_{V}\in {\mathbb{R}}^{L\times d_{v}}$, and compute fused features $F_{fused\;}$. Step 8: Apply Attention Pooling to $F_{fused\;}$ to get $f_{\mathrm{attn}}$, and Max Pooling to get $f_{\max}$. Step 9: Concatenate poolings: $f_{\mathrm{pool}\;}=\left[f_{\mathrm{attn}\;}\;f_{\max}\right]\in {\mathbb{R}}^{2L}$. Step 10: Input $f_{pool}$ into the Softmax function to construct the output $\hat{y}$. Step 11: end Step 12: return the trained model.

In the proposed framework, Transformer-based models capture long-range dependencies, while BiLSTM-based approaches are effective for short-term dependencies. The proposed method integrated the long-range and short-term features. Compared to attention-based fusion methods that use self-attention within a single modality, this cross-attention mechanism enables bidirectional information flow between global and local feature spaces, allowing each branch to benefit from the other’s perspective. The proposed architecture with cross-attention fusion preserves the integrity of both feature types and learns their interactions more effectively.

## 3. Experiment Results and Analysis

### 3.1. Experimental Setup and Parameter Setting

**Figure 2:**
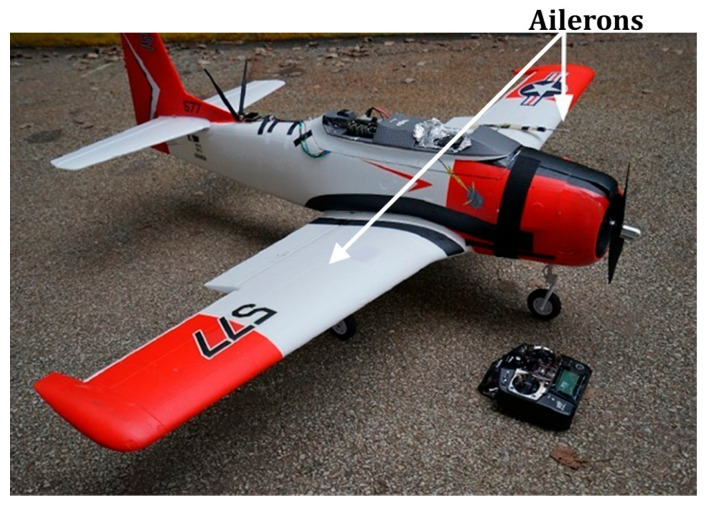
Carbon-ZT-28 fixed-wing UAVs.

To verify the effectiveness of the proposed method, the airLab failure and anomaly (ALFA) dataset from Carnegie Mellon University in the United States is employed [24]. This dataset is based on the Carbon-ZT-28 fixed-wing UAV, as illustrated in Figure 2. Detailed data can be found at: http://theairlab.org/alfa-dataset (accessed on 10 October 2025).

Choose data from the ALFA dataset for four different scenarios: fault-free, left aileron jamming, right aileron jamming, and bilateral aileron jamming scenarios. Each type of dataset contains data from two flight tests. The training set consists of data from one flight experiment, while the validation and test sets are constructed from the other separate flight experiment. Due to variations in flight durations across experiments, the exact number of samples differs per fault category. In terms of approximate sample distribution, the training set accounts for about 60% of the total samples, the validation set about 10%, and the test set about 30%.

To address the issue of limited faulty data, we implement lightweight enhancement strategies on both the training and test sets. Calculate the minimum and maximum values for normalization, as well as the variance of the noise, using the training set. We add Gaussian noise to the original signals and introduce random proportional scaling to simulate fluctuations in UAV flight systems. To ensure the diagnosis performance of the designed system, we subject these data to min-max normalization, mapping the amplitude to the [0, 1] interval to reduce the impact of dimensional differences on training performance. The data augmentation techniques include amplitude scaling (with a range of 0.95–1.05) and adding additive Gaussian noise with variance estimated from the training data. These specific values are chosen to increase the diversity of the training set and prevent overfitting.

Although the validation set only contains approximately 97–98 samples per fault class, it is important to note that each sample is a window of 1024 consecutive residual points. This window length preserves the temporal and frequency characteristics of the fault signals, ensuring that each sample contains enough information to extract both global and local fault features. The experimental configuration and main hyperparameters are listed in Table 1.

After the data enhancement, calculate the residual signals r(k) using the UAV model and measurement value for each scenario. The VMD algorithm requires careful parameter selection to effectively separate the multi-modal coupled residual signals. We configure the VMD with a number of modes H=4, a penalty parameter α=2000, and a convergence tolerance $\varepsilon =1\times 10^{-7}$ for VMD. These parameters are determined through a systematic grid search and domain knowledge. Additionally, set the number of hidden units in the BiLSTM layer to 128 and 256, respectively. Moreover, the Transformer model comprises 2 encoder layers with 4 attention heads, and the feedforward layer dimension is 256. The cross-attention module uses a N=4 heads mechanism, and Query/Key/Value dimension is 256. After determining the parameters of the fault diagnosis model, the four IMFs of VMD are used as input for training the model.

### 3.2. Fault Diagnosis Results and Analysis

**Figure 3:**
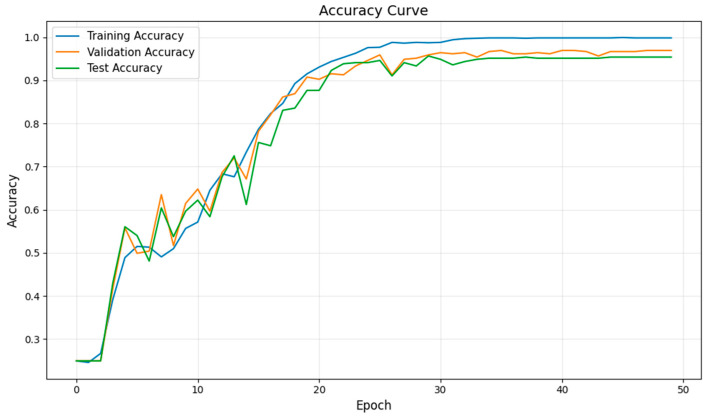
The loss curve of VMD + Transformer + BiLSTM method during the training process.

**Figure 4:**
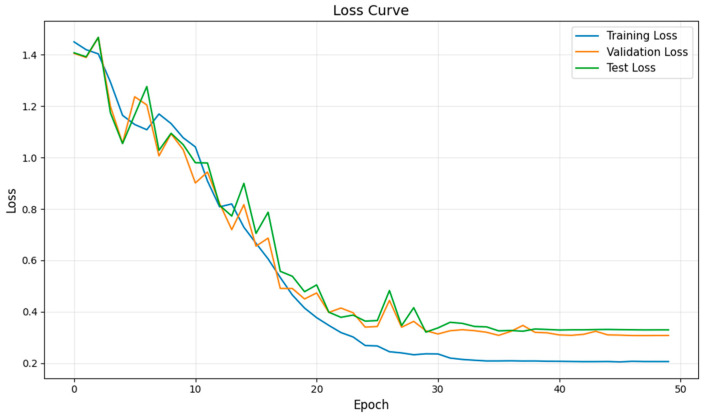
The accuracy curve of VMD + Transformer + BiLSTM method during the training process.

**Figure 5:**
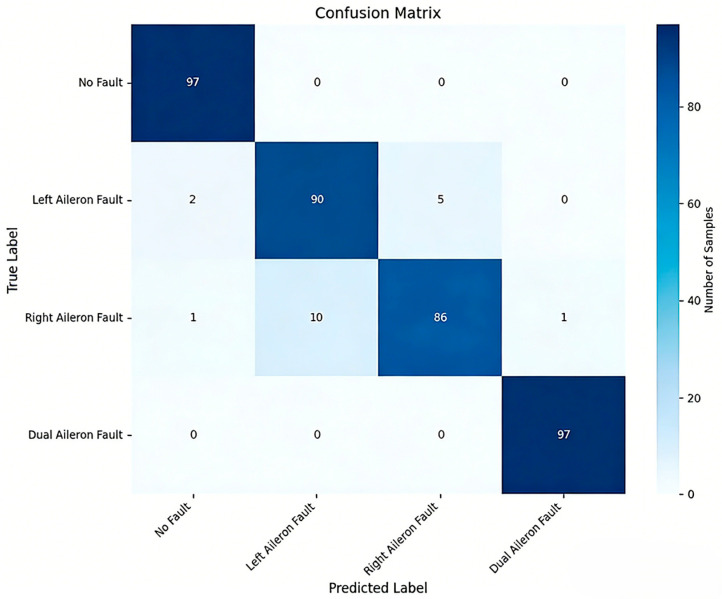
Confusion matrix of VMD + Transformer + BiLSTM method.

**Figure 6:**
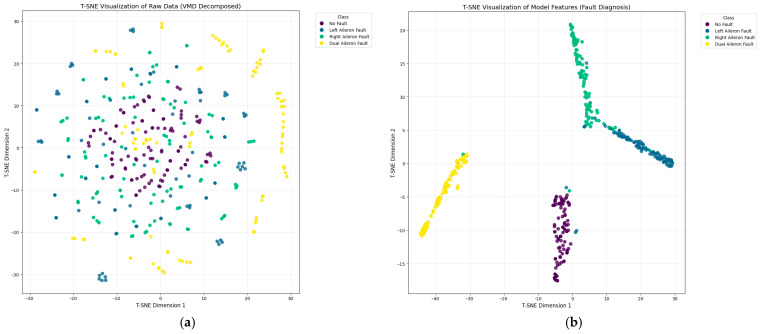
Visualization of the data feature distribution using t-SNE: (**a**) original data feature, (**b**) extracted data feature.

After determining the parameters of the fault diagnosis model, the four IMFs of VMD are used as input for training the model. During the training process, changes in loss value, and accuracy are shown in Figure 3 and Figure 4. The final confusion matrix is shown in Figure 5. To visualize the features of the original data and those extracted by the model, the t-distributed stochastic neighbor embedding (t-SNE) algorithm is utilized for dimensionality reduction, as presented in Figure 6a and Figure 6b, respectively.

From Figure 3, it is evident that the fault diagnosis model initially has a high loss value and low accuracy. However, as the number of iterations increases, the loss value decreases rapidly and stabilizes around the 30th iteration. Similarly, the accuracies of the training and test sets also increase and stabilize after approximately 30 iterations, indicating that the diagnosis model has converged. Additionally, the trends of the key performance indices for both the training and test processes are consistent, with no signs of overfitting. These results confirm the stability and robustness of the proposed model with the selected parameters.

Figure 5 shows that the proposed method has an overall fault detection rate of 98.97%, which is significantly higher than the value of 93.83% in [19]. The diagnosis accuracy is 95.12%. The recall values for fault-free, left, right, and dual aileron faulty cases are 100.00%, 92.78%, 87.56%, and 100.00%, respectively. The precision values for these cases are 97.00%, 90.00%, 94.51%, and 98.98%, respectively. The F1 scores for these cases are 98.50%, 91.39%, 91.13%, and 99.49%, respectively. The average recall, precision, and F1 scores are 95.13%, 95.12%, and 95.13%, respectively. However, the majority of false diagnoses occur between the left and right aileron faults. Specifically, more than half of the misclassifications involve the right aileron fault being identified as a left aileron fault, while some misclassifications involve left aileron faults being misclassified as right aileron faults. This is due to the opposite deflection directions of the left and right ailerons, but their vibration transmission paths, moment responses, and displacement amplitudes tend to be symmetric in UAV flight control systems.

Figure 6a shows that the raw features are mixed together. In Figure 6b, residual signals from the same case exhibit similar features in the two-dimensional space, while signals from different cases have distinct characteristics with limited overlaps. These results demonstrate that the extracted features of different fault types form well-separated clusters, indicating that the model has successfully learned to distinguish between mechanically symmetric faults (left vs. right aileron) based on subtle differences in residual dynamics. The proposed framework effectively learns fault features from VMD-processed residuals without relying on manual thresholding.

### 3.3. Comparative Experiments

**Figure 7:**
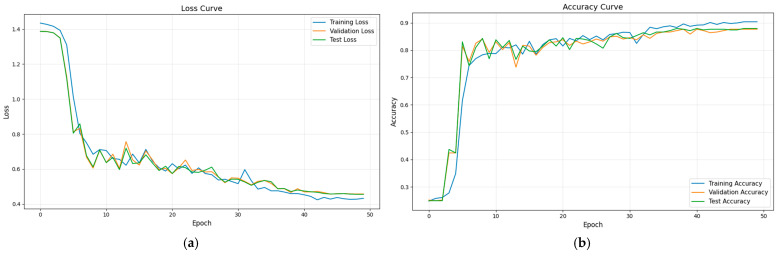
The parameter curves of VMD +BiLSTM method during the training process: (**a**) loss curve, (**b**) accuracy curve.

**Figure 8:**
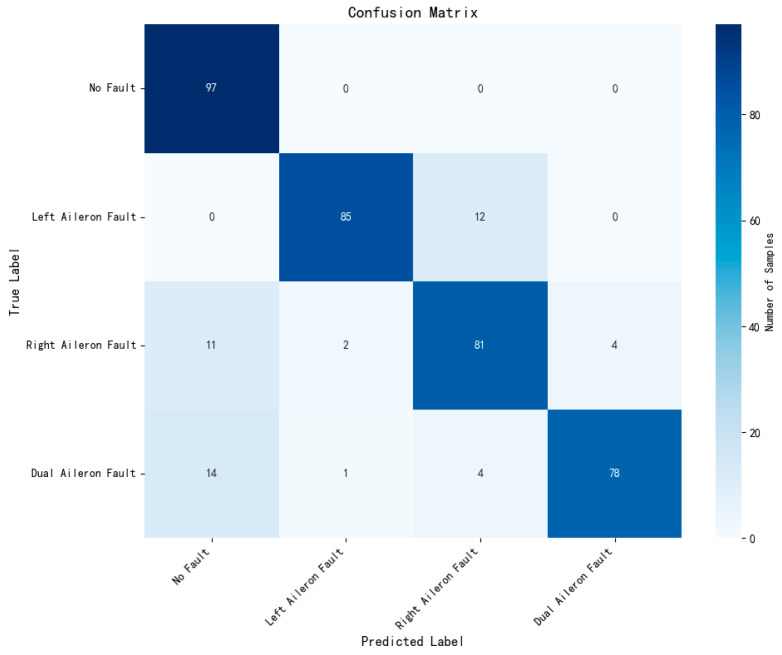
Confusion matrix of VMD + BiLSTM method.

**Figure 9:**
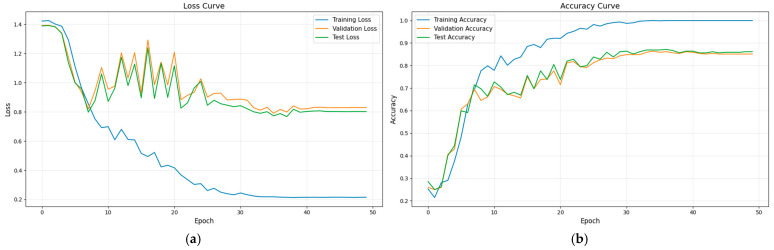
The parameter curves of VMD + Transformer method during the training process: (**a**) loss curve, (**b**) accuracy curve.

**Figure 10:**
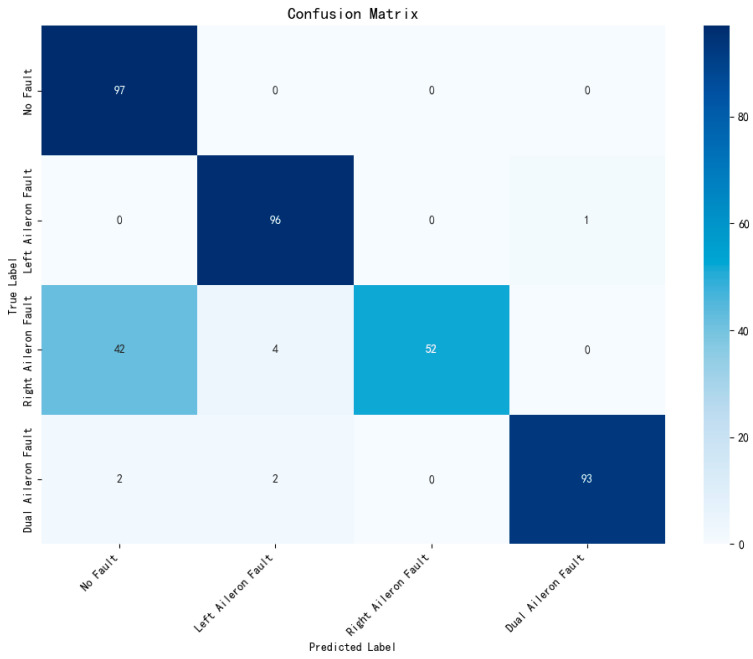
Confusion matrix of VMD + Transformer method.

**Figure 11:**
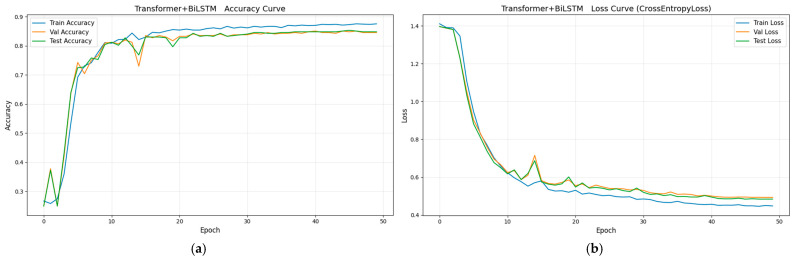
The parameter curves of Transformer + BiLSTM method during the training process: (**a**) loss curve, (**b**) accuracy curve.

**Figure 12:**
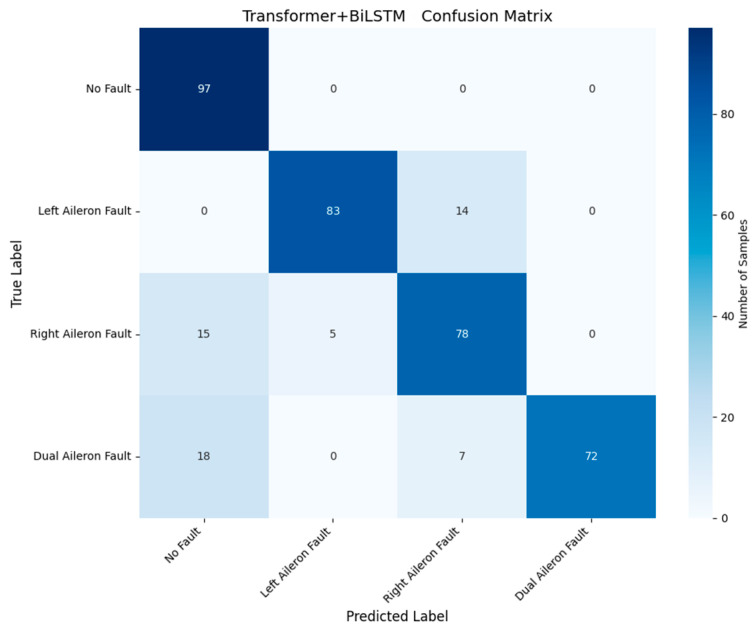
Confusion matrix of Transformer + BiLSTM method.

**Figure 13:**
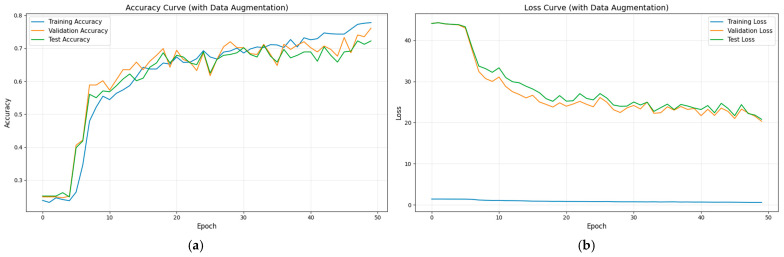
The parameter curves of Transformer method during the training process: (**a**) loss curve, (**b**) accuracy curve.

**Figure 14:**
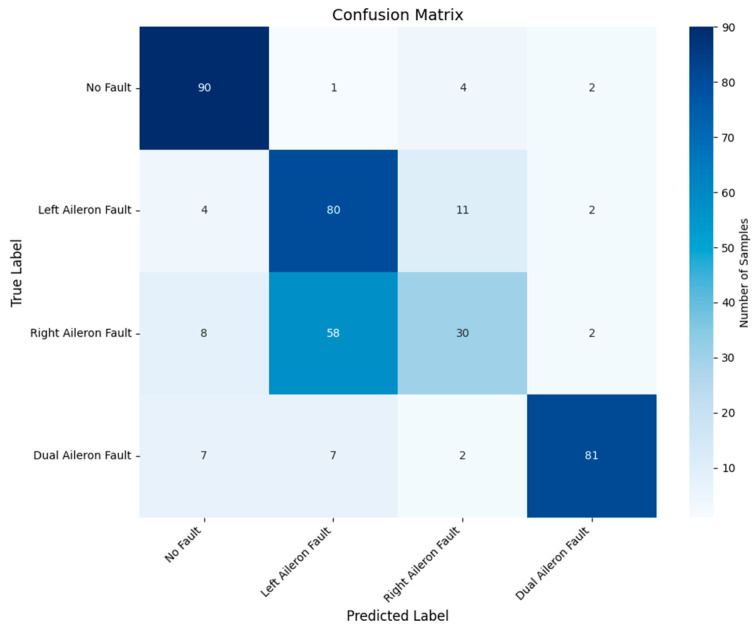
Confusion matrix of Transformer method.

**Figure 15:**
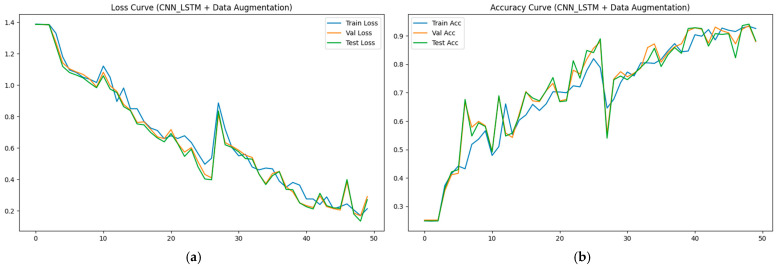
The parameter curves of CNN + LSTM method during the training process: (**a**) loss curve, (**b**) accuracy curve.

**Figure 16:**
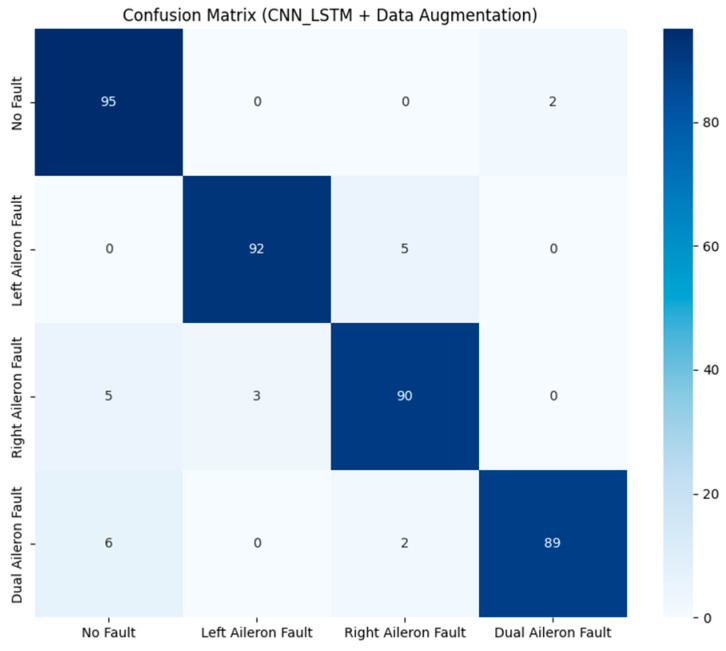
Confusion matrix of CNN + LSTM method.

**Figure 17:**
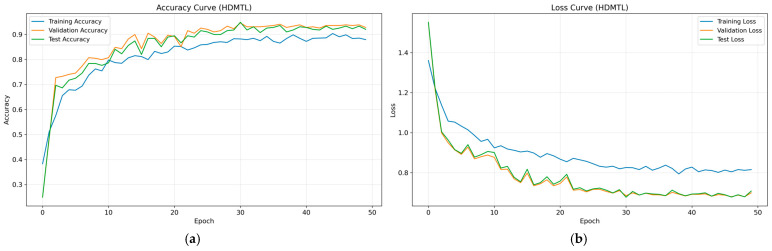
The parameter curves of HDMTL method during the training process: (**a**) loss curve, (**b**) accuracy curve.

**Figure 18:**
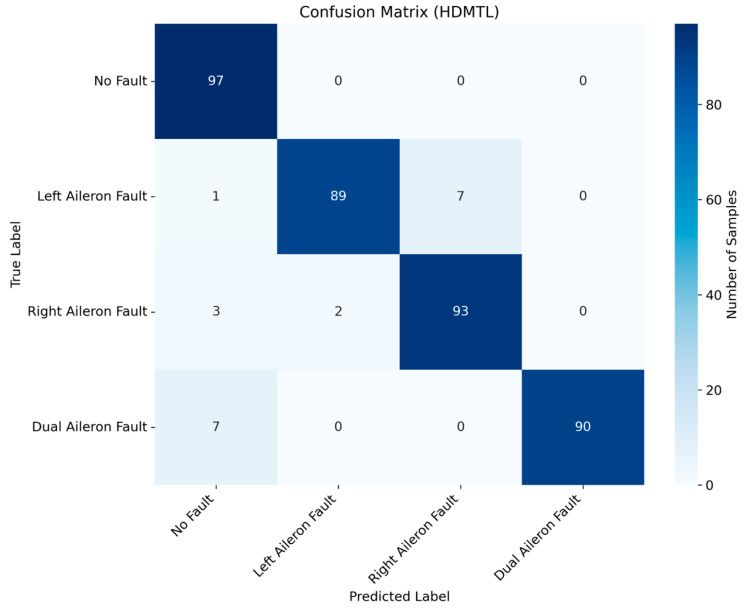
Confusion matrix of HDMTL method.

To validate the superiority of the proposed method in aileron fault diagnosis, we conduct comparative experiments, including VMD + BiLSTM [17], VMD + Transformer [18], Transformer + BiLSTM, pure Transformer, CNN-LSTM, and HDMTL (an attention-based strategy) [22]. All comparison methods were implemented on the ALFA dataset with the same experimental settings described in Section 3.1. The average value of five experiments was used as the final result. The performance metrics of the comparative algorithms are presented in Figure 7, Figure 8, Figure 9, Figure 10, Figure 11, Figure 12, Figure 13, Figure 14, Figure 15, Figure 16, Figure 17 and Figure 18. Table 2 lists the accuracy, average recall, average precision, and average F1-score of the proposed scheme and the comparative experiments.

Figure 7 and Figure 8 demonstrate that the VMD + BiLSTM-based diagnosis method has an accuracy of 87.66%, with average recall, precision, and F1 scores of 87.67%, 88.68%, and 88.18%, respectively. Similarly, Figure 9 and Figure 10 show that the VMD + Transformer method has an accuracy of 86.89%, with average recall, precision, and F1 scores of 86.98%, 90.46%, and 88.18%. However, all of these metrics are lower than those of the proposed method, indicating that the combination of VMD and a single structure (Transformer or BiLSTM) struggles to fully extract local dynamic change features.

Figure 11 and Figure 12 demonstrate that the Transformer + BiLSTM-based diagnosis method achieves an accuracy of 84.83%, with average recall, precision, and F1 scores of 84.85%, 87.20%, and 86.02%, respectively. Similarly, Figure 13 and Figure 14 show that the pure Transformer method achieves an accuracy of 72.24%, with average recall, precision, and F1 scores of 72.34%, 73.57%, and 72.96%, respectively. Compared with the proposed VMD + BiLSTM + Transformer method, these results show that the VMD method can effectively extract the distinct fault features for left and right aileron faults with similar vibration transmission paths.

Figure 15 and Figure 16 demonstrate that the CNN + LSTM method achieves an accuracy of 94.05%, with average recall, precision, and F1 scores of 94.09%, 94.26%, and 94.18%, respectively. However, these metrics are lower than those of our proposed method, indicating that the CNN + LSTM-based diagnosis performance is inferior. Additionally, Figure 17 and Figure 18 display the main parameters for the HDMTL method, another attention-based diagnosis approach. This method achieves an accuracy of 94.86%, with average recall, precision, and F1 scores of 94.86%, 95.15%, and 95.01%. While the accuracy, recall, and F1 scores are below those of our proposed scheme, the precision is slightly higher. Overall, the performance of the HDMTL method is slightly inferior to the proposed method.

These experimental results suggest that relying solely on the structure of Transformer, VMD + BiLSTM, VMD + Transformer, or BiLSTM + Transformer is insufficient for fully extracting global and local fault features. Additionally, the diagnosis performance of the proposed scheme is superior to that of CNN + LSTM and HDMTL. In comparison, our method, which integrates VMD, BiLSTM, Transformer, and attention-based structures, achieves better performance metrics. The proposed feature fusion mechanism effectively extracts comprehensive features of residuals under different scenarios, leading to improved fault diagnosis performance. These results confirm the effectiveness and superiority of our proposed fault diagnosis model.

### 3.4. Extended Validation Results

The training and validation sets used in this study were constructed from different flight experiments within the ALFA dataset. This approach introduces variations in flight states, environmental noise levels, and operational conditions. The results show that the model trained on data collected under specific conditions performs reliably on data acquired under different flight states or noise environments, demonstrating its inherent generalization capability. This robustness can be attributed to the VMD pre-processing, which extracts frequency features robust to noise, and the cross-attention fusion mechanism, which focuses on discriminative fault patterns.

**Figure 19:**
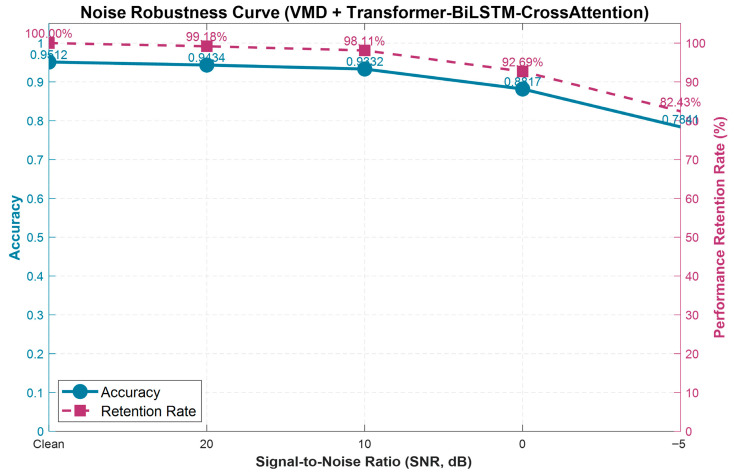
Noise robustness analysis of the proposed method.

As illustrated in Figure 19, the framework shows clear degradation as noise levels increase. In a clean environment, the model achieves an accuracy of 95.12%. However, with additional noise at a signal-to-noise ratio (SNR) of 20 dB, the accuracy only decreases slightly to 94.34%, with a performance retention rate of 99.18%. Even with moderate noise at 10 dB, the accuracy only drops by 0.2%, and the performance remains at 98.11%. However, when the noise level reaches 0 dB, meaning that the noise and effective signal strength are equal, the data becomes severely contaminated. Despite this, the model still maintains an accuracy of 88.17% and a performance retention rate of 92.69%. In the extreme case of −5 dB, where the noise is stronger than the effective signal, it is highly unlikely to encounter such harsh environments in reality. In this scenario, the model still achieves a respectable accuracy of 78.41% and a performance retention rate of 82.43%. These results demonstrate the framework’s robust performance across a range of noise levels, indicating its potential applicability in complex real-world environments. Future work could involve validating the framework on additional platforms as more fixed-wing UAV fault datasets become available.

## 4. Conclusions

In this paper, a hybrid fault diagnosis framework has been proposed for aileron faults in fixed-wing UAV flight control systems. The framework addresses the challenges of strong noise, multi-modal coupling, and limited fault samples. It combines VMD for multi-scale decomposition, Transformer for global feature extraction, and BiLSTM for local temporal modeling in a dual-branch architecture. The main innovation of this work is the feature fusion mechanism, specifically designed to distinguish mechanically symmetric aileron faults. The cross-attention module, in particular, effectively integrates global and local fault signatures, making it a significant improvement over existing hybrid models. Extensive experiments on the ALFA dataset showed that the proposed method achieved 95.12% accuracy, outperforming other methods such as VMD + BiLSTM (87.66%), VMD + Transformer (86.89%), Transformer + BiLSTM (84.83%), Transformer (72.24%), CNN + LSTM (94.05%), and HDMTL (94.86%). The results confirm the contribution of each module and demonstrate the reliability of the proposed framework for fixed-wing UAV aileron fault diagnosis. It has potential applications in flight safety monitoring and predictive maintenance.

Despite these promising results, several limitations of this method should be indicated. The framework has only been validated on the ALFA dataset, and its generalization to other UAV platforms or fault types needs to be verified. The integration of Transformer encoders, BiLSTM networks, and cross-attention fusion results in significant computational overhead, which may limit the application on resource-constrained UAV platforms. The performance also depends on critical hyperparameters, such as the number of VMD modes, Transformer layers, and attention heads. While grid search was used for parameter selection, a more systematic optimization approach is needed to avoid degrading model performance.

Future work will focus on addressing the limitations of the current framework. To improve generalization, the framework should be extended to include other UAV components and fault types. Additionally, incorporating additional sensor modalities such as motor current and GPS data will provide a more comprehensive state monitoring system. To reduce computational costs, more efficient and lightweight models can be explored for online diagnosis. The automated hyperparameter optimization techniques can also be investigated to enhance adaptability. Furthermore, self-supervised learning techniques can be integrated to enable fault diagnosis without labeled data and to interpret anomalies by identifying which flight variables deviate from normal patterns. The development of digital twin systems for UAVs offers a promising solution to the data scarcity problem. Finally, the framework will be extended to handle unknown fault types, which will significantly enhance its practical utility.

## Figures and Tables

**Table 1:** Experimental configuration and hyperparameters.

Component	Parameter	Value/Setting
Data Preprocessing	Time step per sample L	1024
	Data augmentation	Amplitude scaling [0.95, 1.05], Gaussian noise
VMD	Number of modes H	4
	Penalty parameter α	2000
	Convergence tolerance	10^−7^
Transformer	Number of encoder layers	2
	Number of attention heads	4
	Feedforward dimension	256
	Dropout rate	0.3
BiLSTM	Hidden sizes	128,256
Cross-Attention	Number of heads	4
	Query/Key/Value dimension	256
Training	Optimizer	Adam
	Learning rate (max)	10^−3^
	Weight decay	10^−4^
	Batch size	32
	Epochs	50
	Learning rate scheduler	OneCycleLR
	Label smoothing	0.05

**Table 2:** The accuracy, average recall, average precision and average F1-score of different methods.

Approach	Accuracy	Average Recall	Average Precision	Average F1-Score
VMD + Transformer + BiLSTM	95.12%	95.13%	95.12%	95.13%
VMD + BiLSTM	87.66%	87.67%	88.68%	88.18%
VMD + Transformer	86.89%	86.98%	90.46%	88.72%
Transformer + BiLSTM	84.83%	84.85%	87.20%	86.02%
Transformer	72.24%	72.34%	73.57%	72.96%
CNN + LSTM	94.05%	94.09%	94.26%	94.18%
HDMTL	94.86%	94.86%	95.15%	95.01%

## Data Availability

The airLab failure and anomaly (ALFA) dataset can be found at: http://theairlab.org/alfa-dataset (accessed on 10 October 2025).
